# Routine Screening for Sickle Cell Disease during Pregnancy: Epidemiological and Haemoglobin Profile in Congo

**Published:** 2022-03-14

**Authors:** Alexis Elira Dokekias, Josué Simo Louokdom, Letso Thibaut Ocko Gokaba, Firmine Olivia Galiba Atipo Tsiba Gokaba, Jayne Chelsea Bango, Lydie Ngolet Ocini, Clatere Itoua, James Taylor

**Affiliations:** 1 Department of pharmacy Université des Montagnes; 2 Sickle Cell Disease Center du Congo (CNRD); 3 Department of Obstetrics and Gynaecology; 4 Howard University Washington, DC, USA

**Keywords:** Emmel test, Haemoglobin profile, Sickle-cell disease, Pregnancies, Immunochromatography

## Abstract

Sickle-cell disease, a genetic condition with a high prevalence in sub-Saharan Africa, is transmitted in an autosomal recessive mode. Its screening during pregnancy makes it possible to identify carriers of the S gene which constitute a risk for the unborn child. In order to promote the use of immuno-chromatographic tests, we have set ourselves the task of establishing the epidemiological profile and determining the Emmel test performance.

Analytical cross-sectional study of three months duration carried out in the 12 departments of Congo in pregnant women, from 12 weeks of amenorrhea, Admitted for Antenatal Consultation (ANC). The studied variables were epidemiological, Emmel test and immuno-chromatographic profile of haemoglobin.

782 pregnant women screened, of which 27.88% were AS sickle cell trait and 1.79% homozygous SS. The median age of sickle cell patients was 29 years vs. 25 years (p=0.10). High education level, married status, history of transfusion and sickle cell disease, and high ANC number were more common in pregnant sickle cell patients (p<0.05).The frequency of sickle cell trait ranged from 16.67 to 31.17% and homozygous forms from 0 to 66.67% depending on the department. The sensitivity and specificity of the Emmel test were 46% and 99% with PPV and NPV of 95% and 81% respectively.

Sickle cell disease carriage, which is high in both forms, is more often of interest to young, educated, married pregnant women and follow-up by health personnel other than the doctor in rural areas.

## INTRODUCTION

Sickle cell disease, a genetic condition of high prevalence in sub-Saharan Africa, results from the presence of a mutated allele of the globin gene. The A allele is replaced by an S allele which codes for an abnormal Haemoglobin (Hb), Hb S [1–3]. Patients with homozygous SS sickle cell disease receive from both parents this allele or the S allele associated with another variant, including Hb C and beta-thalassemia. When these parents are heterozygous, they have 25% risk of transmitting the disease to the child at each conception. Testing during pregnancy makes it possible to identify S gene carriers which pose a risk to the unborn child, inform parents, offer genetic counseling for subsequent pregnancies, and plan neonatal screening. Moreover, a pregnancy in a known sickle cell patient is a high maternal-fetal risk situation. Given that the prevalence of sickle-cell disease is high in Congo [4,5], screening for it during pregnancy is mandatory. Given the weakness of the technical equipment system, particularly in rural areas, this screening is most often done with the Emmel test. However, in the literature, there are an increasing number of easy-to-use POCT tests that allow a more efficient diagnosis. Thus, in order to promote the use of Point of Care Testing (POCT) in Congo, we proposed to establish the epidemiological and haemoglobin profile of sickle cell pregnant women and determine the diagnostic performances of the Emmel test.

## PATIENTS AND METHODS

It was an analytical cross-sectional study carried out from 5th January to 30th March 2020 in the 12 departments of Congo (Bouenza, Brazzaville, Cuvette Centrale, Cuvette Ouest, Kouilou, Lekoumou, Likouala, Niari, Plateaux, Pointe Noire, Pool, and Sangha). It concerned all pregnant women of black race, from 12 Weeks of Amenorrhoea (WA), Admitted for Antenatal Consultation (ANC) in the Integrated Health Centres (IHC) or Reference Hospitals of the capitals of each department. Those from Brazzaville were received at the Sickle Cell Center, Centre National de Reference de la Drepanocytose (CNRDr) in Congo. The informed consent of each pregnant woman was obtained before and after an information session. Pregnant women who did not have an Emmel test result were not included. For each pregnant woman, we studied sociodemographic variables (age, marital status, occupation, level of education); medical and obstetrical variables (history of sickle cell disease, blood transfusion, gestational age, parity, pregnancy follow-up) and biological variables (Emmel test and immunochromatographic profile of haemoglobin). Consecutive sampling was carried out over the study period.

The results of the Emmel test were obtained from prenatal consultation records or hospital laboratory records. For the immuno-chromatographic test, 5 μl of blood was taken from the terminal end of the index finger using a pro-pipette under aseptic conditions. The blood samples were analyzed extemporaneously using the Sickle Scan® screening kit from Biomedomics laboratories. The results were compared with the results of the Emmel.

Data entry and analysis were done using Microsoft Excel version 17.2 and R 3.6.3. Qualitative variables were presented as numbers and percentage, while quantitative variables were presented as a mean and its standard deviation. The comparison of the variables of interest was done with the Chi^2^ and anova tests. The diagnostic performance of the Emmel test was evaluated by intrinsic and extrinsic characteristics. All tests were performed at the threshold α=5%.

## RESULTS

Seven hundred and eighty-two women were screened; including 218 cases of sickle cell trait AS and 14 cases of homozygous form SS, being 27.88 and 1.79% respectively. The median age of sickle cell patients was 29 [26-31] vs. 25 [20-32] years (p-value=0.10). Sickle cell pregnancies were better educated (p-value=0.009) than other pregnancies (Table 1). Similarly, history of sickle cell disease and red blood cell transfusion were significantly more frequent in those with sickle cell disease (Table 1). The distribution of sickle cell disease in pregnant women by department is reported in Table 2. The sensitivity and specificity of the Emmel test were 0.46 [0.39-0.52] and 0.99 [0.97-0.99] with positive and negative predictive values of 0.95 [0.89-0.98] and 0.81 [0.78-0.84] respectively (Table 3).

## DISCUSSION

The objective of this study was to establish the epidemiological, obstetrical and haemoglobin profile of sickle cell pregnancies in Congo. The haemoglobin phenotype was established by immuno-chromatography. This method allows a qualitative identification of hemoglobins A, S and C with a sensitivity and specificity comparable to that of electrophoresis techniques [6,7]. At the end of our work, we obtained high prevalences of the heterozygous (27.88%) and homozygous (1.79%) forms. On the other hand, they are lower in West and East Africa with 0.14 to 0.20% of homozygous forms and 11.11 to 19.77% of heterozygous forms [8–10]. The location of the Republic of Congo in the epicenter of the Sickler belt [1,11], the superposition of the S gene distribution map and that of malaria could explain the high prevalences observed in our study. Indeed, in order to better resist malaria, which is endemic in this geographical area, the populations of this region, like those in other areas of high malaria endemicity, have undergone mutations that have led to the appearance of the S gene. Subjects carrying this mutation in its heterozygous form show some resistance to Plasmodium falciparum [11,12]. Selection pressure over the years would have contributed to the increased frequency of the S gene. Furthermore, the high prevalence of homozygous forms is more often attributed to consanguinity [13], the lack of knowledge of the disease by the population, especially on the mode of transmission. Information, education, communication and mandatory systematic screening before marriage are strategies to reduce the prevalence of homozygous forms of the disease [14]. The above measures would help reducing the prevalence of this condition, and anticipating the care of women of childbearing age.

The average age of pregnant women with sickle-cell disease, similar to that of Galiba et al. in Congo and Rajab in the Middle East, is higher than that of Muganyizi in Tanzania. This difference would be explained by the selection criteria of the study population [14–16].

Our study reports like Nwabuko in Nigeria (2016) that the pregnant sickle cell patient is better educated than other pregnant women (p-value=0.009). Indeed, the education of the sickle cell woman would play an important role in her procreation plan. It would enable sickle-cell women to be more responsible in their life choices, which would also justify the fact that they are more married than the other pregnant women in this study (p-value=0.038). The education of sickle-cell women should be promoted because education and responsibility are necessary for efficient participation in the care process.

As for ANC, the mean number is low for a gestational age of 24 ± 8 WA in sickle cell pregnancies. This age is relatively similar to that reported by Nwabuko and Onohau Nigeria [9,17]. Moreover, 28.57 per cent of women with sickle cell disease found in rural areas did not see a doctor during prenatal consultations. This raises not only the problem of access to quality health care for women with sickle-cell disease, but also the problem of strengthening the capacities of midwives in the management of pregnancies among women with sickle-cell disease and the organization of multi-disciplinary consultations (gynaecologist, haematologist and pediatrician) in order to improve the monitoring of pregnant women. Indeed, a pregnancy evolving in a sickle cell patient being a high-risk situation [16,18–21], the ANC interval should be shorter to optimize obstetrical management. In addition, antenatal care is one of the pillars of the safe motherhood initiative to prevent adverse pregnancy outcomes.

In terms of technical performance, the Emmel test is a diagnostic orientation test for sickle cell disease, which consists of searching *in vitro* sickle cells in the absence of oxygen. Our study reported a low sensitivity compared to the results of immunochromatography. Our observations are in agreement with those of Diallo et al. in Mali [10]. These results show the need to popularize points of care testing which, in addition to being less expensive compared to reference techniques (HPLC and electrophoresis), do not require equipment or highly qualified personnel. Moreover, their sensitivities and specificities are comparable to those of alkaline pH electrophoresis techniques.

## CONCLUSION

Carriage of hemoglobinosis S is elevated in both forms in Congolese pregnant women. They are most often young, educated, married and do not meet any doctor during ANC in rural areas. Several challenges remain to be addressed with a view to improve the obstetrical prognosis, which is potentially fraught with stillbirths and obstetrical morbidity. These challenges require the implementation of a national screening strategy using reliable methods and capacity building of health personnel.

## Figures and Tables

**Table 1:** Distribution of pregnant carriers of haemoglobin (Hb) S and AA according to epidemiological characteristics between January and March 2020.

	Hb SS	Hb AS**	Hb AA	Total	p-value
	n=14 (%)	n=218 (%)	n=550 (%)	n=782 (%)	
Age groups					0.713
[15-17]	0 (0.00)	14 (6.42)	44 (8.00)	58 (7.42)	
[18-24]	3 (21.43)	88 (40.37)	218 (39.64)	309 (39.51)	
[25-31]	7 (50.00)	59 (27.06)	161 (29.27)	227 (29.03)	
[32-45]	4 (28.57)	57 (26.15)	127 (23.09)	188 (24.04)	
Marital status					0.038
Single*	11 (78.57)	200 (91.74)	519 (94.36)	730 (93.35)	
Married	3 (21.43)	18 (8.26)	31 (5.64)	52 (6.65)	
Education level					0.009
Unschooled	0 (0.00)	15 (6.88)	36 (6.55)	51 (6.52)	
Primary	0 (0.00)	45 (20.64)	125 (22.73)	170 (21.74)	
Secondary	11 (78.57)	140 (64.22)	369 (67.09)	520 (66.50)	
Tertiary	3 (21.43)	18 (8.26)	20 (3.64)	41 (5.24)	
Profession					0.273
Informal	10 (71.43)	162 (74.31)	439 (79.82)	611 (78.13)	
Formal	1 (7.14)	29 (13.30)	53 (9.64)	83 (10.61)	
Student	3 (21.43)	27 (12.39)	58 (10.55)	88 (11.25)	
Hb SS in the family	9 (64.29)	33 (15.14)	33 (6.00)	75 (9.59)	<0.001
Transfusion	12 (85.71)	29 (13.30)	87 (15.82)	128 (16.37)	<0.001
Weeks of amenorrhea					
median [q1-q3]	25 [21-29]	26 [20-31]	25 [20-32]	26 [20-32]	0.96
[37-39]	1 (8.33)	9 (4.37)	32 (6.43)	42 (5.87)	0.655
[40-43]	0 (0.00)	6 (2.91)	20 (4.02)	26 (3.63)	
Pregnancy follow-up					
Not followed up	1 (7.14)	12 (5.50)	26 (4.73)	39 (4.99)	
Midwife	4 (28.57)***	187 (85.78)	498 (90.55)	689 (88.11)	
Doctor	9 (64.29)	7 (3.21)	4 (0.73)	20 (2.56)	
Other	0 (0.00)	12 (5.50)	22 (4.00)	34 (4.35)	
Gravida					0.066
Paucigravid	12 (85.71)	131 (60.09)	318 (57.82)	461 (58.95)	
Multigravid	2 (14.28)	87 (39.91)	143 (42.18)	186 (41.05)	
Parity					0.448
Nulliparous	7 (50.00)	60 (27.52)	145 (26.36)	212 (27.11)	
Pauciparous	6 (42.86)	116 (53.21)	298 (54.18)	420 (53.71)	
Multiparous	1 (7.14)	42 (19.27)	107 (19.45)	150 (19.18)	
ANC					
median [q1-q3]	3 [3-4]	1 [1-3]	1 [1-3]	1 [1-3]	0.01
[0-3]	5 (62.50)	130 (87.84)	322 (87.03)	457 (86.88)	0.127
[4-7]	3 (37.50)	18 (12.16)	48 (12.97)	69 (13.12)	

Note:

* Single, Widow, and Free union

**: 4 cases of Hb AC;

***: All living in rural areas ;

ANC: Antenatal consultation.

**Table 2:** Prevalence of haemoglobins (Hb) in pregnant women by departments between January and March 2020.

		Abnormal Hb		Normal Hb	Total
	SS	AS or AC	Total abnormal Hb	AA	
Departments	n (%)	n (%)	n (%)	n (%)	n (%)
Bouendza	2 (1.18)	53 (31.17)*	55 (32.4)	115 (67.65)	170 (21.74)
Brazzaville	10 (66.67)	3 (20.00)	13 (86.7)	2 (13.33)	15 (1.92)
Cuvette Centrale	-	2 (16.67)	2 (16.7)	10 (83.33)	12 (1.53)
Cuvette Ouest	-	24 (32.43)	24 (32.4)	50 (67.57)	74 (9.46)
Likouala	-	17 (36.17)	17 (36.2)	30 (63.83)	47 (6.01)
Kouilou	-	19 (36.54)	19 (36.5)	33 (63.46)	52 (6.65)
Lékoumou	-	8 (22.22)	8 (22.2)	28 (77.78)	36 (4.60)
Niari	-	16 (27.12)	16 (27.1)	43 (72.88)	59 (7.54)
Plateau	1 (1.45)	16 (23.19)	17 (24.6)	52 (75.36)	69 (8.82)
Pointe Noire	1 (1.10)	22 (24.18)	23 (25.3)	68 (74.73)	91 (11.64)
Pool	-	21 (26.25)	21 (26.2)	59 (73.75)	80 (10.23)
Sangha	-	17 (22.08)	17 (22.1)	60 (77.92)	77 (9.85)
Total	14 (1.79)	218 (27.88)	232 (29.67)	550 (70.33)	782 (100)

Note:

* Hb AC: 2.35 %.

**Table 3:** Emmel diagnostic test performance.

Immunochromatography test				
		Hemoglobin S	Hemoglobin AA	Total
Emmel test	Hemoglobin S	107	5	112
	Hemoglobin AA	125	545	670
	Total	232	550	782

Se=0.46 (0.39-0.52) ; Sp=0.99 (0.97-0.99)

PPV=0.95 (0.89-0.98) ; PNV=0.81 (0.78-0.84)

## Abbreviations:

ANC: Admitted for Antenatal Consultation
Hb: Haemoglobin
ESRD: End Stage Renal Disease
POCT: Point of Care Testing
CNRDr: Centre National de Reference de la Drepanocytose
IHC: Integrated Health Centres
WA: Weeks of Amenorrhoea
DSMB: Data Safety Management Board
